# Comparison of metagenomic next-generation sequencing and conventional culture for the diagnostic performance in febrile patients with suspected infections

**DOI:** 10.1186/s12879-024-09236-w

**Published:** 2024-03-26

**Authors:** Hui Yang, Nannan Xu, Meichen Yan, Lulu Yang, Sai Wen, Shanshan Wang, Chunmei Qu, Ke Xu, Xuying Yang, Gang Wang

**Affiliations:** 1 Department of Infectious Disease, Qilu Hospital, Cheeloo College of Medicine, Shandong University, Jinan, Shandong 250012 China; 2 Department of Intensive Care Unit, Qilu Hospital, Cheeloo College of Medicine, ShandongUniversity, Jinan, Shandong 250012 China; 3 Department of Scientific Affaires, Hugobiotech Co., Ltd., No.1, East Disheng Road, Beijing, 100176 China

**Keywords:** Metagenomic next-generation sequencing, Conventional culture, Febrile patients, Infectious diseases, Diagnostic performance

## Abstract

**Background:**

Timely and accurate identification of pathogens is crucial for appropriate treatment and prognosis of infectious diseases. As an increasingly popular pathogen detection method, the performance of metagenomic next-generation sequencing (mNGS) in detecting pathogens in febrile patients with suspected infection requires further exploration.

**Methods:**

This study included 368 febrile patients with suspected infections who were admitted to the Infectious Disease Department of Qilu Hospital, Shandong University between January 5, 2021 and April 14, 2023. Both mNGS testing and conventional culture were performed in all patients. Clinical data of enrolled patients were collected, and the diagnostic performances of mNGS and culture were compared.

**Results:**

Of the 368 enrolled patients, 231 were finally diagnosed with infection and 137 were with diseases other than infection. The sensitivity (58.01% *vs.* 21.65%, *p* < 0.001) and negative predictive value (54.67% *vs.* 42.9%) of mNGS were superior to those of culture. In contrast, the culture exhibited higher specificity (99.27% *vs.* 85.40%, *p* < 0.001) and positive predictive value (98.84% *vs.* 87.01%) than mNGS. Among infected patients with positive mNGS results, 64 received adjusted antibiotic therapy including treatment transitions, antibiotic downgrading, and combination therapy. Among them, 9 had additional antifungal drugs and 21 patients had a treatment turning point based on the mNGS results and these patients recovered and discharged due to timely antibiotic adjustment. Both positive rates of puncture fluid mNGS and tissue mNGS were higher than those of culture in the patients who had prior antibiotic use, and this difference was statistically significant (*p* = 0.000).

**Conclusion:**

mNGS is more sensitive and accurate than traditional culture, making it ideal for identifying pathogens and screening infectious diseases, especially for those with uncultivated or difficult-to-cultivate species. Early diagnosis allows for prompt treatment with targeted antibiotics, and mNGS is recommended when samples are limited.

**Supplementary Information:**

The online version contains supplementary material available at 10.1186/s12879-024-09236-w.

## Introduction

Fever refers to the body’s temperature that is higher than the normal range due to an increase in set-point temperature in the hypothalamus. Common causes of fever include infections, autoimmune disorders, hematological abnormalities, neoplasms, and unidentified factors. Distinguishing between infectious and non-infectious diseases is crucial in febrile patients, as unidentified infections can lead to delayed or insufficient treatment, prolonged hospital stays, recurrent hospitalizations, heightened mortality rates, and increased disability rates [1]. A study conducted by *Rudd et. al*. revealed that infections account for over 20% of global deaths, establishing it as one of the foremost causes of mortality on a global scale [2, 3], especially in cases with sepsis which results in approximately 5 million deaths per year worldwide. Early identification and aggressive treatment of infection is critical for patient survival and prognosis [4, 5].

Pathogens play a pivotal role in the development of infectious diseases, and comprehending their etiology is imperative for effective disease management and ultimate outcome. Presently, diagnostic techniques for identifying pathogens encompass culture, serological tests, pathological examinations, and pathogen sequencing [6]. Serological tests and pathological tests are convenient and expeditious but not suitable for all pathogens. Culture-based methods offer the advantage of broad applicability across a diverse spectrum of pathogens, enabling the assessment of drug sensitivity and resistance. Nevertheless, the time-intensive nature of culture, typically spanning 1–5 days, may delay treatment initiation, especially for slow-growing microorganisms, such as fungi and mycobacteria. Moreover, the positive rate of culture is notably diminished in individuals with prior antibiotic exposure [7], and not all pathogens can be acquired using conventional culture techniques. Conventional pathogen detection methods are inadequate to meet the clinical need. Metagenomic next-generation sequencing (mNGS) is an emerging molecular diagnostic approach and offers several advantages, such as rapid detection, independence from antibiotic influence, and the capability to identify clinically uncommon, challenging-to-cultivate, and novel pathogens [8, 9]. Nonetheless, it is costly, susceptible to host DNA interference, and necessitates specialized equipment, proficient technicians, and extensive bioinformatics expertise. The interpretation of results poses a significant challenge [10, 11].

Presently, the existing research on the effectiveness of conventional culture and mNGS diagnostics primarily relies on limited sample sizes, specific sample types, or infection sites [12–18]. There is a notable dearth of comprehensive studies encompassing diverse sample types, and the clinical utility of mNGS and conventional culture in the context of infectious diseases remains subject to evaluation. In order to assess the merits and drawbacks of these two diagnostic approaches, the present study gathered mNGS and culture data from febrile patients with suspected infections to assess the diagnostic performance of different detection methods.

## Methods

### Study objects

This retrospective analysis gathered clinical data from patients who admitted to the Infectious Diseases Department of Qilu Hospital, Shandong University due to fever and suspected infection between January 5, 2021, and April 14, 2023. The ultimate diagnosis of patients was ascertained through retrospective review by three proficient infectious disease physicians based on the patient's medical history, diagnostic tests, imaging examinations, pathological findings, and treatment outcomes. The comprehensive dataset comprises various essential variables, such as patient demographics (gender, age, admission date, height, weight, and Body Mass Index, BMI), clinical diagnosis and medical history, serum albumin levels, sample type, detection results and duration of culture and mNGS, as well as antibiotic usage and modifications.

### Inclusion criteria

The inclusion criteria for patient enrollment are as follows: (1) patients exhibiting fever (≥ 37.3 °C) and suspected infections upon admission who underwent both conventional culture and mNGS procedures; (2) patients with a time interval of less than 24 h between the collection of the same sample type for culture and mNGS; (3) complete mNGS and culture were conducted for sample types, including blood, puncture fluid, tissue, bronchoalveolar lavage fluid (BALF), and cerebrospinal fluid (CSF).

### Exclusion criteria

Patients were excluded from the study if they met any of the following criteria: (1) patients with a definitive infection diagnosis upon admission and a confirmed pathogen; (2) individuals with incomplete mNGS or culture data, or a time lapse exceeding 24 h between mNGS and culture; (3) cases with mNGS and culture conducted using samples, such as sputum or urine; (4) patients with incomplete clinical data.

### Conventional microbiological culture

Samples including blood, CSF, BALF, puncture fluid, and tissues were processed in the microbiology laboratory. Positive culture specimens were identified using matrix-assisted laser desorption ionization-time of flight mass spectrometry (MALDI-TOF). The VITEK II compact system was used for drug sensitivity testing and AST-GN334, AST-GN335 and AST-GP639 drug sensitivity cards were used to determine the minimum inhibitory concentration (MIC). Antibiotic susceptibility testing was performed in accordance with the guidelines outlined by Clinical and Laboratory Standards Institute (CLSI).

### Metagenomic next-generation sequencing

Blood samples were transported to the laboratory within a temperature range of 6 °C to 35 °C. Other fluid samples were aseptically sealed, stored at -20 °C, or transported using dry ice to the testing laboratory. A 200 µL volume of sample was utilized for DNA extraction and purification using QIAamp DNA Micro Kit (QIAGEN, Hilden, Germany) following the instructions. The concentration and quality of the DNA were assessed using the Qubit 3.0 fluorometer (Invitrogen, Q33216) and agarose gel electrophoresis (Major Science, UVC1-1100). DNA library was constructed using Qiagen's QIAseq Ultralow Input Library Kit (QIAGEN, Hilden, Germany) according to the guidelines. The quality of the libraries was evaluated using the Qubit 3.0 fluorometer (Invitrogen, Q33216) and the Agilent 2100 Bioanalyzer (Agilent Technologies, Palo Alto, USA). The qualified DNA libraries were sequenced on the Illumina Nextseq 550 platform (Illumina San Diego, USA).

Adapters, low-quality, low-complexity, and short sequences were removed from the raw data after sequencing. Subsequently, the SNAP software was employed to eliminate human sequences based on human reference database (hg38). The remaining data were then aligned against microbial genome databases using the Burrow-Wheeler Alignment. The microbial composition within the samples was analyzed to identify the pathogens.

### Result interpretation criteria

Positivity for mNGS/culture was defined as a positive result obtained from mNGS/culture, and were confirmed as causative pathogens by clinicians. Conversely, negativity was defined as the absence of any pathogen detected by mNGS/culture. Contamination was defined as the pathogens were detected by mNGS/culture, which were not consistent with clinical diagnosis, and had not been clinically confirmed, and was not considered to be the pathogen of clinical disease. Given the inability of conventional culture to capture viral pathogens, this study specifically concentrated on comparing bacterial and fungal findings. Viruses detected by mNGS were not included in the further analysis.

### Statistical methods

Statistical analysis was performed using SPSS 25.0 software. Paired sample T-tests were utilized for comparing continuous variables between two groups, while the chi-squared test or Fisher's exact test were employed for categorical variable comparisons. The clinical diagnosis was considered as the gold standard, and McNemar's test was employed to compare the sensitivity and specificity of paired mNGS and culture. Kappa consistency tests were utilized to evaluate the consistency between the two diagnostic methods. Logistic regression was applied to calculate odds ratios and their corresponding 95% confidence intervals. A *p*-value of less than 0.05 was defined as statistically significant.

## Results

### General clinical characteristics

**Fig. 1:**
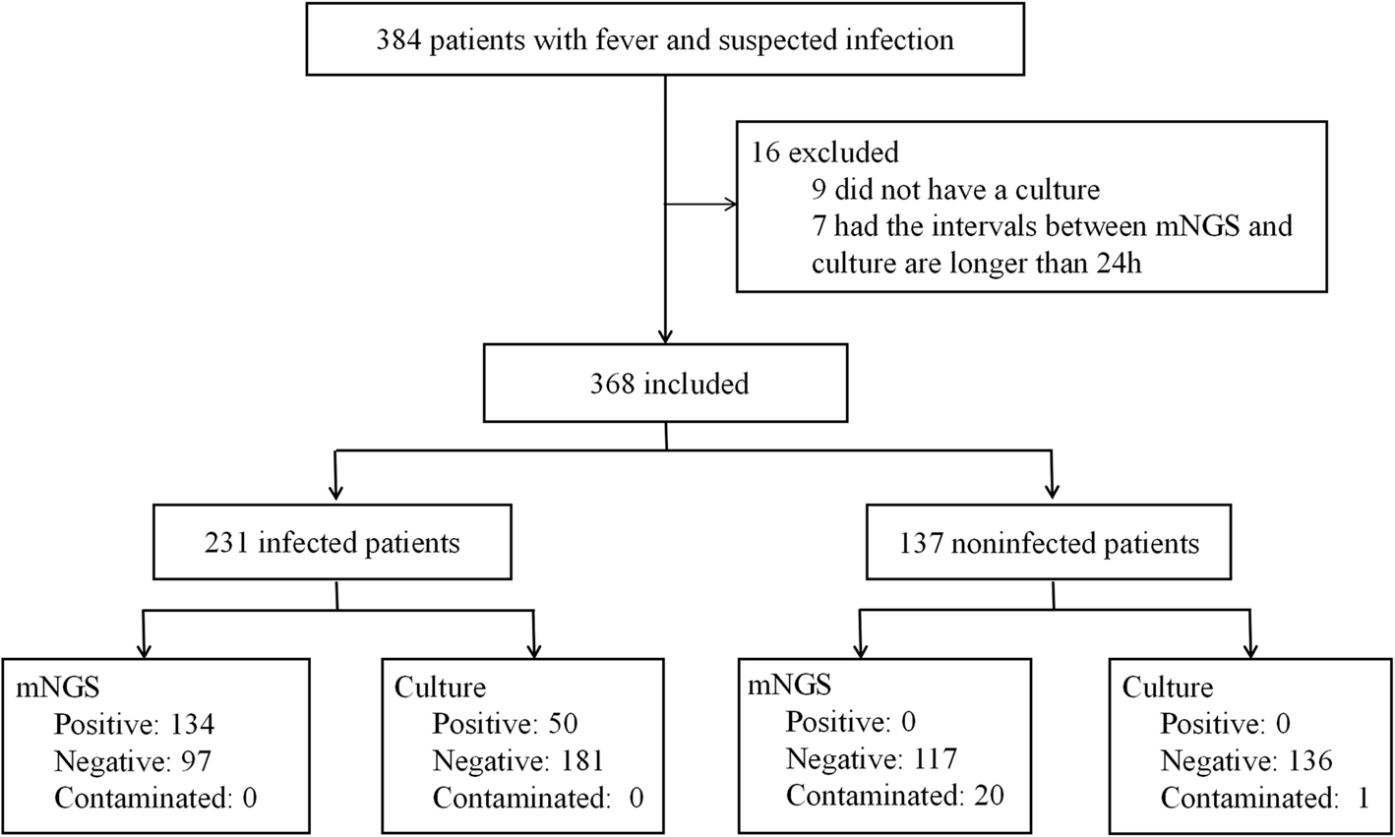
Flowchart of febrile patients with suspected infections enrolled

**Fig. 2:**
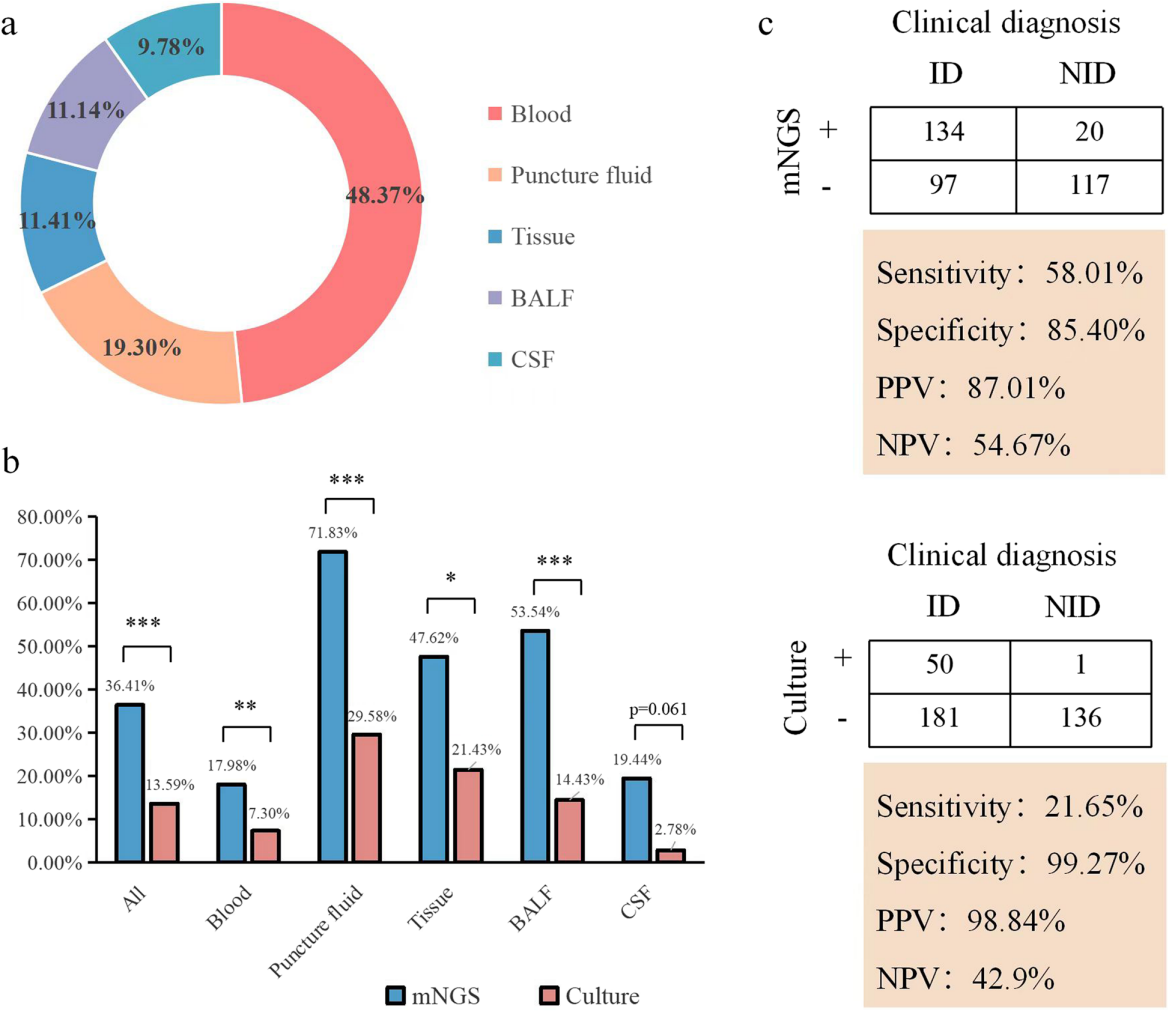
Comparison of diagnostic performance between mNGS and culture. **a** Percentage of different sample types; **b** The histogram for omparison of positive rate between mNGS and culture in different sample types; **c** The four-grid table of diagnostic performance for mNGS and culture using clinical diagnosis as the gold standard

A total of 368 patients were eventually included in this retrospective study (Fig. 1). The majority of specimens consisted of blood samples (48.37%), followed by puncture fluid (19.30%), tissue (11.41%), BALF (11.14%), and CSF (9.78%) (Fig. 2a). The median age of patients was 57 years (range: 10–92), and 215 were male (58.40%). The 231 patients (62.78%) were finally diagnosed with infectious diseases, while 137 (37.22%) had diseases other than infections. The rates of comorbidity for diabetes, cardiovascular diseases, and obesity in patients with infections were higher than that in non-infected patients. The 58.05% of infected patients were aged more than 60 years, significantly higher than non-infected patients (32.12%) (chi-squared test, *p* = 0.003). Some of these febrile patients with suspected infections were diagnosed with "unknown cause of fever" upon admission, while others were diagnosed with "consciousness disorders", "unknown cause of fever after valve replacement surgery", "unknown cause of back pain", and so on. The distribution of infection sites varied, with bloodstream infections accounting for the highest proportion (27.71%, 64/231), followed by pulmonary infections (23.38%, 54/231). Other sites of infection encompassed the central nervous system, liver, spinal joints, skin soft tissues, muscles, etc. Detailed demographic information is presented in Table 1.

**Table 1:** General demographic information

Characteristics	Noninfectious diseases (137)	Infectious diseases (231)	*p*
**Gender**			
Male	76 (55.47%)	139 (60.17%)	
Female	61 (44.53%)	92 (39.83%)	
**Age (years)**			0.003
< 60	93 (67.88%)	120 (51.95%)	
≥ 60	44 (32.12%)	111 (58.05%)	
**Hypoproteinemia**	29 (21.17%)	68 (29.44%)	0.088
**Obesity (BMI ≥ 28)**	10 (7.30%)	23 (9.96%)	0.039
**Chronic disease**			
Diabetes	15 (10.95%)	56 (24.24%)	0.002
Cardiovascular disease	33 (24.09%)	86 (37.23%)	0.011
Cerebrovascular disease	10 (7.30%)	14 (5.91%)	0.667
Chronic kidney disease	1 (0.73%)	10 (4.33%)	0.059
Chronic respiratory disease	1 (0.73%)	2 (0.87%)	1.000
Connective tissue disease	7 (5.11%)	10 (4.33%)	0.799
Chronic liver disease	5 (3.65%)	7 (3.03%)	0.768
**Affected sites**			
Bloodstream	/	64	
Liver	/	29	
Urogenital system	/	7	
Endocardium	/	13	
Blood vessel	/	7	
Spine and joints	/	28	
Lung	/	54	
Skin, soft tissues, and muscles	/	26	
Abdomen	/	20	
CSF	/	34	
Lymphnode	/	5	
Endophthalmitis	/	1	

### Diagnostic performance of mNGS vs. culture

The overall positive rate of mNGS was 36.41% (134/368). The positive rates varied by sample type, with puncture fluid having the highest rate at 71.83%, followed by BALF (53.54%), tissue (47.62%), CSF (19.44%), and blood (17.98%). In contrast, the overall positive rate of culture was 13.59% (50/368), with puncture fluid culture had the highest positive rate (29.58%), followed by tissue (21.43%), BALF (14.43%), blood (7.30%), and CSF (2.78%) (Fig. 2b). Puncture fluid consistently had the highest positive rate by both mNGS and culture. Additionally, the contamination rate of mNGS (5.43%) was higher than that of culture (0.27%), with the most common contaminants being *Propionibacterium acnes*, *Staphylococcus hominis*, and *Corynebacterium*.

In the infectious diseases group, the mNGS results were positive for 134 patients (58.01%, 134/231), while the culture results were positive for 50 patients (21.65%, 50/231). In the non-infectious diseases group, mNGS showed 117 negative (85.40%, 117/137) and 20 contaminated results (14.60%, 20/137); culture showed 136 negative (99.27%, 136/137) and 1 contaminated results (0.73%, 1/137). Overall, the sensitivity, specificity, positive predictive value (PPV), and negative predictive value (NPV) of mNGS were 58.01%, 85.40%, 87.01%, and 54.67%, respectively. The sensitivity, specificity, PPV, and NPV of culture were 21.65%, 99.27%, 98.84%, and 42.9%, respectively. The sensitivity and NPV of mNGS were higher than those of conventional culture (McNemar's test: 58.01% vs. 21.65%, *p* < 0.001; 54.67% vs. 42.9%). Conversely, the specificity and PPV of culture were higher than those of mNGS (McNemar's test: 99.27% vs. 85.40%, *p* < 0.001; 98.84% vs. 87.01%) (Fig. 2c). The diagnostic performance of different samples were shown in Supplementary Table 1. 

The average reporting time of mNGS in our hospital was 24 h (range: 20–36 h), significantly shorter than the results with the initial blood culture and genus identification time frame by MALDI-TOF among the 50 infected patients (45.28 h, ranging from 22 to162 hours), with a *p* value that is 0.000 (T-tests).

### Consistency between mNGS and culture

**Fig. 3:**
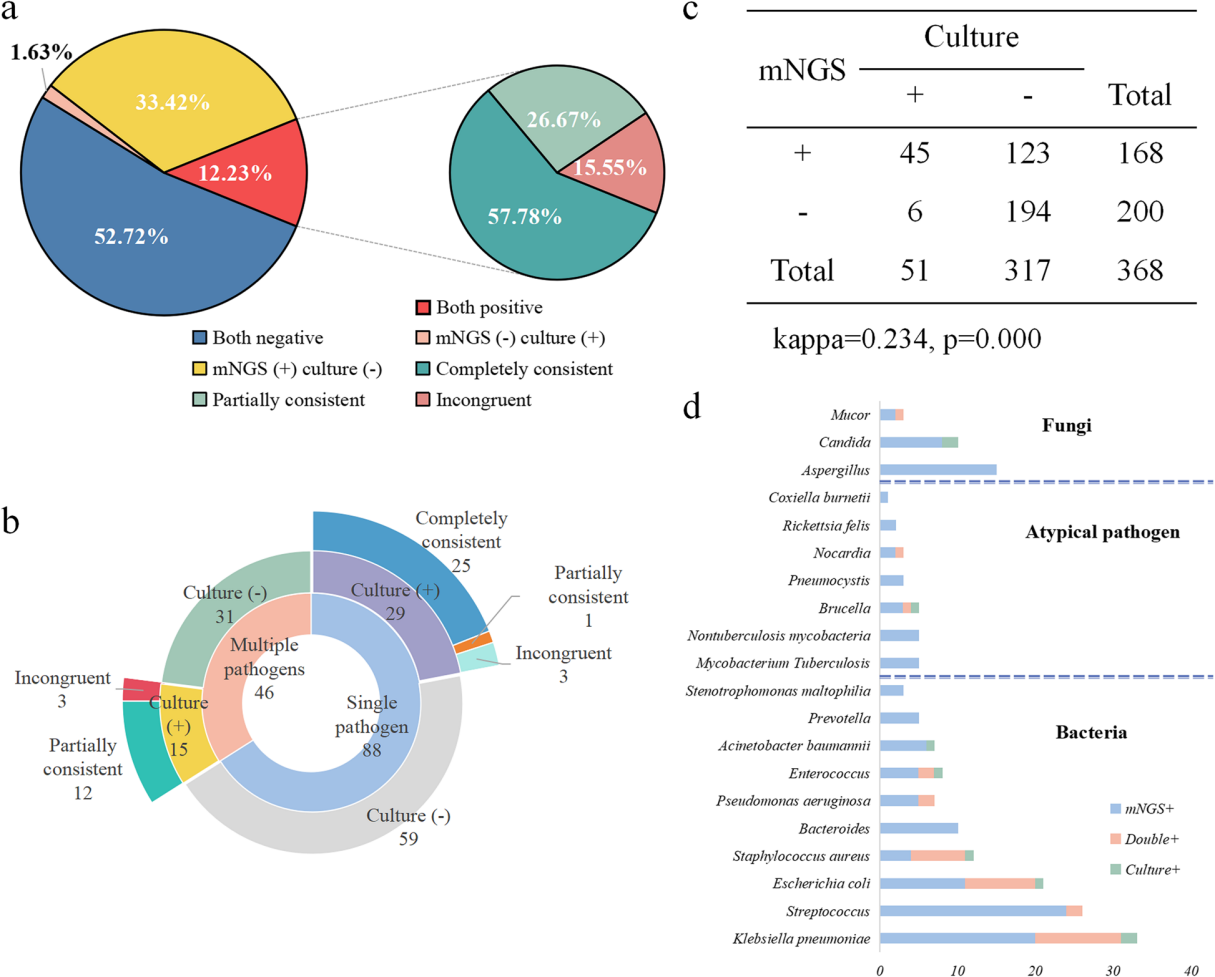
The consistency between mNGS and culture. **a** The consistency ratios of mNGS and culture testing; **b** Rose diagram of the concordance of pathogens detection by different methods; **c** The consistency appraisal of mNGS with culture; **d** The frequency distribution of pathogens spectrum detected by mNGS and culture

In our study, mNGS and culture were both positive in 45 patients (12.23%, 45/368). Among these cases, mNGS and culture showed completely matched results in 26 patients, partial consistency in 12 patients, and complete inconsistency in 7 patients. Conversely, a total of 194 cases (52.72%, 194/368) yielded negative results by both tests. Additionally, 123 cases (33.42%, 123/368) were positive in mNGS but negative in culture, while 6 cases (1.63%, 6/368) were positive in culture but negative in mNGS (Fig. 3a). Details are outlined in Table 2. The agreement between positive mNGS and culture results among patients with infection was relatively low, with a kappa value of 0.234 (Kappa consistency tests) (Fig. 3b).

**Table 2:** Comparison of mNGS and culture consistency in different sample types

Sample type	mNGS ( +) culture ( +)	mNGS ( +) culture (-)	mNGS (-) culture ( +)	mNGS (-) culture (-)	Total
All	45 (12.23%)	123 (33.42%)	6 (1.63%)	194 (52.72%)	368
Blood	10 (5.62%)	43 (24.16%)	4 (2.24%)	121 (67.98%)	178
Puncture fluid	21 (29.58%)	31 (43.66%)	0	19 (26.76%)	71
Tissue	7 (16.67%)	17 (40.48%)	2 (4.76%)	16 (38.09%)	42
BALF	6 (14.63%)	21 (51.22%)	0	14 (34.15%)	41
CSF	1 (2.78%)	11 (30.55%)	0	24 (66.67%)	36

Among the 231 patients with infectious diseases, 88 were identified to have single pathogen infection by mNGS, while 46 patients were detected with infections of multiple pathogens by mNGS. Among the 88 patients with a single pathogen identified through mNGS, 29 were also culture positive, with 25 cases showing concordance between culture and mNGS results. Three cases exhibited complete inconsistency, and one patient's blood culture yielded two distinct pathogens. Among the 46 patients with multiple pathogens detected by mNGS, 15 were culture positive. Among these, three cases demonstrated complete inconsistency between mNGS and culture, while the remaining 12 patients had all their cultured pathogens within those identified by mNGS (Fig. 3c).

The most common bacteria identified by mNGS were *Klebsiella pneumoniae* (*n* = 33), *Streptococcus* (*n* = 26), *Escherichia coli *(*n* = 20), *Staphylococcus aureus* (*n* = 11), and *Enterobacter* (*n* = 10). As*pergillus*, *Candida*, and *Mucor* were the predominant fungi. Furthermore, there were also atypical pathogens detected by mNGS, including *Mycobacterium tuberculosis*, *non-tuberculous mycobacteria* (NTM), *Brucella*, *Pneumocystis*, *Nocardia*, *Chlamydia*, and *Coxiella*. The most frequently detected bacteria by conventional culture were *K. pneumoniae* (*n* = 13), *E. coli* (*n* = 10), *S. aureus* (*n* = 8), *Enterococcus* and *Streptococcus*. Few atypical pathogens and fungi were detected by culture (Fig. 3d).

### Modification of antibiotic treatments based on mNGS

Among the cohort of patients with infections, a total of 134 cases yielded positive results for mNGS. Of these cases, 64 patients experienced adjusted antibiotic treatments based on mNGS testing. This modification encompassed an escalation in antibiotic usage for 41 patients, discontinuation of one or two antibiotics for 2 patients, and a complete alteration in the antibiotic regimen for 21 patients (Table 3). Notably, among patients with adjusted antibiotics, 14 were identified to have single or multiple anaerobic bacteria, such as *Bacteroides fragilis*, *Prevotella*, and *Parvimonas micra*. As a result, the utilization of anaerobic bacterial treatment in clinical therapy was enhanced. Furthermore, mNGS prompted the prescription of antifungal drugs for 9 patients and 21 patients had a treatment turning point because of mNGS results.The pathogens detected in these instances frequently posed difficulties in cultivation or eluded identification through conventional culture techniques, encompassing *Brucella*, *Pneumocystis jirovecii*, NTM, *M. tuberculosis*, *Coxiella burnetii*, *Rickettsia felis*, and *Aspergillus*. These patients recovered and discharged due to timely antibiotic adjustment.

**Table 3:** Strategies of antibiotic change

Antibiotic strategy	Patient number (%)
Add 1 agent	30 (22.39%)
Add 2 agents	6 (4.48%)
Add 3 agents	2 (1.49%)
Add 4 agents	3 (2.23%)
Remove 1 agent	1 (0.75%)
Remove 2 agents	1 (0.75%)
Change completely	21 (15.67%)
No change	70 (52.24%)

This study encompassed a cohort of 71 cases with puncture fluid samples and 42 cases with tissue samples. With the exception of 5 cases where tissue samples were procured via surgical procedures, the remaining samples were acquired under the guidance of ultrasound or computed tomography(CT). Among the total of 113 puncture fluid and tissue samples, 15 cases were categorized as non-infection cases, while the infection group comprised 98 cases, as determined by the final clinical diagnosis. Among the 98 infected patients, 6 had not been administered antibiotics before mNGS collection, while 92 had a recent history of antibiotic use prior to collection (Supplementary Fig. 1).There were 92 infected patients with a recent history of antibiotic use, the positive rate of mNGS was 75.00%, significantly higher than that of culture (28.26%, *p* = 0.000).

## Discussion

Pathogens play a crucial role in the etiology of infectious diseases, and timely identification of these pathogens is instrumental in determining appropriate infection management strategies, thereby impacting disease outcomes. Presently, conventional cultures and mNGS are common techniques for pathogen identification. This retrospective study aimed to comprehensively assess the diagnostic efficacy of conventional cultures and mNGS in diagnosing febrile patients with suspected infections. The analysis encompassed 368 cases across five different specimen types, with the objective of offering improved guidance for selecting diagnostic methods in clinical practice.

Across all patients, the positive rate of mNGS was 36.41%, which was lower than previously reported [15, 19, 20]. This discrepancy can be attributed to the specific definition of mNGS positivity employed in our study, which required the identification of pathogens through mNGS and subsequent clinical confirmation. The methodology employed in this study diverges from categorizing all instances of pathogen detection as positive outcomes, thereby resulting in a lower positivity rate when compared to previous investigations. Among infected patients, the positive rate of mNGS was 58.4%, while conventional culture yielded a positive rate of 21.6%. The results were the similar as in previous research [12, 15, 21]. There are several reasons for the higher positive rate of mNGS. Firstly, mNGS serves as an impartial diagnostic approach that identifies DNA/RNA information within specimens through sequencing techniques, regardless of the pathogen activity level. Secondly, most of our patients have previously been hospitalized and given antibiotics, which can reduce the positive rates of culture [7, 22]. Lastly, infectious diseases do not always lend themselves to conventional culture methods, such as fastidious bacteria, anaerobic bacteria and pneumocystis [15, 23].

In the present study, the sensitivity of mNGS was determined to be 58.01%, surpassing that of culture (21.65%). Conversely, culture demonstrated a specificity of 99.27%, which exceeded that of mNGS. These observations align with previous research [12, 19]. The NPV of mNGS was 54.67%, higher than that of culture. When conventional cultures are unable to definitively exclude infection, mNGS can be a supplementary or parallel test to detect the pathogens. In this study, 137 patients were finally diagnosed as non infectious diseases based on their negative mNGS and negative culture results, as well as the judgment of clinicians combined with clinical data. The definitive diagnosis reduced unnecessary antibiotic use, thereby reducing antibiotic related adverse reactions and antimicrobial resistance [24].

Among 231 infected patients in this study, the most frequently detected bacteria by culture are *K. pneumoniae*, *E. coli*, and *S. aureus*, aligning with the findings reported in the Lancet's 2022 publication [3]. The prevalence of fungal infections has been on the rise in recent years especially in immunocompromised individuals with the growing population of solid organ or haematopoietic stem cell transplantation (HSCT) and the increasing number of patients who suffered the AIDS without timely treatment. Additionally, the incidence of NTM is rapidly increasing, posing a considerable public health concern. Thus, mNGS has become a valuable adjunct in identifying fungi and NTM [25–27]. In this study, mNGS prompted modifications to antibiotic strategies in 64 patients, a total of 43 patients had their antibiotic dosages adjusted based on mNGS detection, including treatment transitions, antibiotic downgrading, and combination therapy. These positive mNGS identified pathogens include pathogens difficult to cultivate such as *Bacteroides fragilis*, *Prevotella*, *Nocardia*, *Aspergillus*, etc., promoting the transition from empirical therapy to targeted therapy. It also included common bacteria such as *Escherichia coli*, *Klebsiella pneumoniae*, and *Staphylococcus aureus*, reducing unnecessary antibiotic combinations and promoting appropriate antibiotic use, and 21 patients experienced a therapeutic turning point as a result of the mNGS results. The identified causative agents in these cases predominantly included *Brucella*, *Pneumocystis*, NTM, *M. tuberculosis*, *Streptococcus pneumoniae*, *Bartonella*, *Rickettsia*, and *Aspergillus*. Notably, mNGS exhibited a higher sensitivity compared to culture in detecting these atypical or challenging-to-culture pathogens [12, 14, 28].

Moreover, mNGS offers expedited outcomes, with an average reporting duration of 24 h, enabling early clinical diagnosis and treatment. This holds significant importance for septic individuals, as timely implementation of targeted antimicrobial therapy is imperative for improving outcomes and reducing mortality [5, 29]. From another perspective, although early bacterial identification of positive specimens could be achieved for culturing positive patients through MALDI-TOF, the average time was still longer than mNGS, and the difference was statistically significant. mNGS had advantages in terms of detection duration, sensitivity, and guidance for early clinical treatment, but it lacked the sensitivity and resistance of antibiotics in vitro, resulting in missing drug sensitivity data and enzyme production data. It could assist in the selection of the right antibiotics, but to some extent, it might not necessarily be able to select the most appropriate antibiotics.

The positive rate and sensitivity of mNGS and culture using puncture fluid in this study were the highest among different sample types. One of the reasons lies in that patients received ultrasound or CT scans before puncture, which indicated localized abscesses or infectious/inflammatory lesions to some extent. In some cases, patients may present with severe infections requiring specialized medical interventions, such as ultrasound or CT-guided aspirations and surgical specimen collection. These samples often have limited quantities and cannot be repeatedly acquired. Furthermore, a significant number of patients receive antibiotic treatment prior to the paracentesis. Currently, less studies have focused on comparing the diagnostic efficiency between mNGS and conventional culture using puncture fluid. Our study findings indicate that in patients with a previous antibiotic usage, both mNGS and culture demonstrated the ability to detect responsible pathogens in specific cases. Nevertheless, the positive rate of mNGS was notably higher than that of culture, and this disparity was statistically significant. These results suggest that the influence of a history of antibiotic use on culture outcomes is more pronounced compared to mNGS. Based on the aforementioned findings, we propose mNGS to detect pathogens when clinical specimens are difficult to collect especially in patients with a history of antibiotic use.

According to Fig. 3a and b, it can be concluded that NGS and culture have relatively low consistency, especially in non-aseptic specimens such as tissue, BALF and puncture fluid. These samples necessitate meticulous analysis of culture and mNGS outcomes. The overall contamination rate of mNGS in this study was 5.43%, higher than that of culture (0.27%). However, there was no statistical significance. Despite rigorous disinfection protocols at the puncture site before blood collection, contamination from skin flora remains unavoidable [1, 30]. It is imperative to incorporate clinical presentation, physical signs, imaging, serology, and even pathological findings to make informed determinations regarding colonization, pathogenicity, and contamination, thereby enabling the selection of the most efficacious antibiotic treatment regimen.

## Limitations

This retrospective study encountered missing data, including height, weight, and paired culture with mNGS, resulting in the exclusion of certain case data from the analysis. The majority of patients had a history of antibiotic usage before specimen collection, leading to a decreased positive rate, particularly for culture. Additionally, the relatively small number of CSF, BALF, and tissue specimens compared to puncture fluid specimens and peripheral blood specimens, posed challenges in conducting subgroup analysis. To better assess the diagnostic performance of mNGS compared to culture, more rigorous prospective studies are needed.

## Conclusion

In this study, mNGS exhibited superior sensitivity and NPV than conventional culture in detecting pathogens. mNGS particularly excelled in identifying microorganisms that are challenging to culture or cannot be cultured, as well as in detecting pathogens in specimens that are difficult to obtain through puncture or intraoperative procedures promoting targeted and precise treatment in clinical practice. mNGS also demonstrated a higher negative exclusion value when screening infectious diseases. However, clinicians should exercise caution and be mindful of colonization, contamination, and pathogenicity when interpreting detected pathogens, given their high sensitivity. While conventional culture can guide antibiotic selection, it has a lower positivity rate and longer culture periods. Therefore, the choice between these methods in clinical practice should be determined by the specific needs of the patient to ensure accurate pathogen diagnosis.

### Supplementary Information

**Additional file 1: Supplementary figure 1.** The diagnostic performance for different methods of puncture fluid and tissue samples after using antibiotics.

## Data Availability

The original data and materials presented in the study are included in the article/supplementary material, further inquiries can be directed to the corresponding author.

## Abbreviations

mNGS: Metagenomic next-generation sequencing
BMI: Body Mass Index
BALF: Bronchoalveolar lavage fluid
CSF: Cerebrospinal fluid
MALDI-TOF: Matrix-assisted laser desorption ionization-time of flight mass spectrometry
MIC: Minimum inhibitory concentration
CLSI: Clinical and Laboratory Standards Institute
PPV: Positive predictive value
NPV: Negative predictive value
NTM: Non-tuberculous mycobacteria
CT: Computed tomography
