# Supplementary material for: Comparison of metagenomic next-generation sequencing and conventional culture for the diagnostic performance in febrile patients with suspected infections
Source: BMC Infect Dis. 2024 Mar 26;24:350. doi: 10.1186/s12879-024-09236-w (PMC10964532; doi:10.1186/s12879-024-09236-w)
Supplement: Supplementary file 1 — Additional file 1: Supplementary figure 1. The diagnostic performance for different methods of puncture fluid and tissue samples after using antibiotics. [file 12879_2024_9236_MOESM1_ESM.docx]

**Supplementary figure 1.** The diagnostic performance for different methods of puncture fluid and tissue samples after using antibiotics.

**
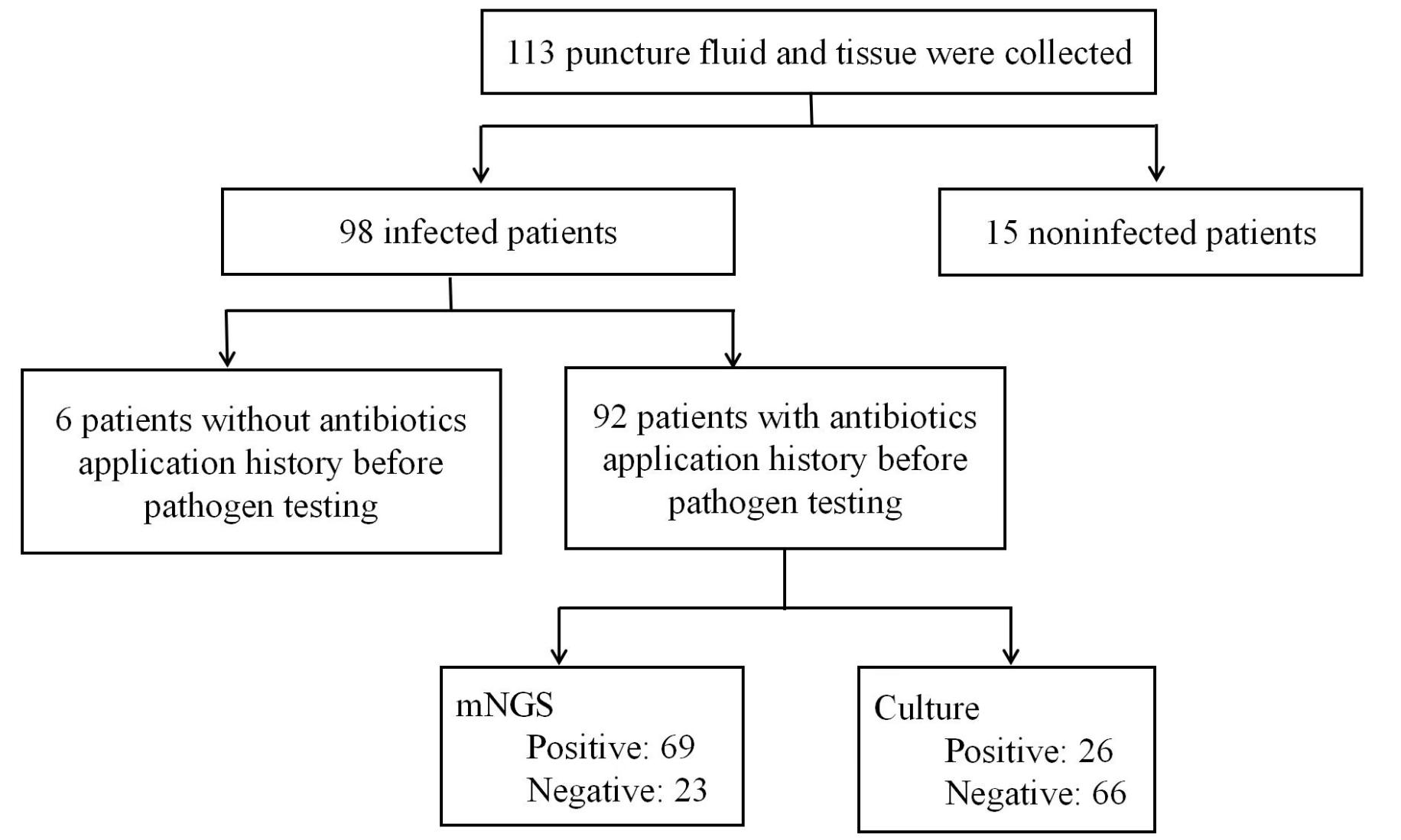
**

**Supplemental table 1.** The diagnostic performance of mNGS and culture in different sample types.

|  | All | | | Blood | | | Puncture fluid | | | Tissue | | | BALF | | | CSF | | |
| --- | --- | --- | --- | --- | --- | --- | --- | --- | --- | --- | --- | --- | --- | --- | --- | --- | --- | --- |
|  | mNGS | culture | *p* | mNGS | culture | *p* | mNGS | culture | *p* | mNGS | culture | *p* | mNGS | culture | *p* | mNGS | culture | *p* |
| Positive rates | 36.41% | 13.59% | 0.000 | 17.98% | 7.30% | 0.004 | 71.83% | 29.58% | 0.000 | 47.62% | 21.43% | 0.021 | 53.54% | 14.43% | 0.000 | 19.44% | 2.78% | 0.061 |
| Sensitivity | 58.01% | 21.65% | 0.000 | 45.07% | 18.31% | 0.001 | 78.46% | 32.31% | 0.000 | 60.61% | 27.27% | 0.006 | 72.73% | 18.18% | 0.000 | 24.14% | 3.45% | 0.052 |
| Specificity | 85.40% | 99.27% | 0.000 | 85.05% | 99.07% | 0.000 | 100% | 100% | 1 | 88.89% | 100% | 1 | 75% | 100% | 0.467 | 85.71% | 100% | 1 |
| PPV | 87.01% | 98.84% | 0.047 | 66.67% | 92.86% | 0.111 | 100% | 100% | 1 | 95.24% | 100% | 1 | 92.31% | 100% | 1 | 87.5% | 100% | 1 |
| NPV | 54.67% | 42.9% | 0.010 | 70% | 64.63% | 0.397 | 30% | 12% | 0.146 | 38.1% | 27.27% | 0.404 | 40% | 22.86% | 0.304 | 21.43% | 20% | 0.889 |
